# Adipose-Derived Mesenchymal Stem Cells, Cell-Based Therapies, and Biomaterials as New Regenerative Strategies in Plastic Surgery

**DOI:** 10.3390/biomedicines10081875

**Published:** 2022-08-04

**Authors:** Pietro Gentile

**Affiliations:** 1 Plastic and Reconstructive Surgery, Department of Surgical Science, “Tor Vergata” University, 00133 Rome, Italy; pietrogentile2004@libero.it; Tel.: +39-3388-5154-79; 2 Academy of International Regenerative Medicine & Surgery Societies (AIRMESS), 1201 Geneva, Switzerland

Adipose-derived mesenchymal stem cells (AD-MSCs), cell-based therapies, and biomaterials are interrelated terms that often go hand in hand when discussing strategies to improve tissue regeneration or to repair tissue defects. Stem cells (mesenchymal and follicular), biotechnologies, blood products (platelet-rich plasma (PRP), and biomaterials (mesh, scaffolds, hydrogels) may be helpful for such applications.

Considering current knowledge on mesenchymal stem cells (MSCs), AD-MSCs, epithelial and dermal cells, human follicle mesenchymal stem cells (HF-MSCs), and biomaterials such as titanium/polypropylene mesh, numerous researchers have developed different strategies to improve the effects of these various biotechnology applications.

This Special Issue aimed to collect multidisciplinary original research articles demonstrating the basic research and clinical impacts of MSCs, AD-MSCs, PRP, micrografts, and biomaterials in tissue repair.

Several fields of study, including neurogenesis [1], dental implant surfaces [2], pancreatic pain [3], neurogenic differentiation [4], polyurethane–fibrin scaffolds [5], chondrogenesis [6], osteoarthritis [7], adipose-derived stem cells [8], and skin photoaging [9] have been considered in the present issue.

The main role of AD-MSCs and fatty tissue has been observed.

In fact, in recent years, surgical procedures in regenerative fields have been deeply modified, with a gradual shift towards less invasive strategies based on autologous fat grafting (FG) and related to AD-MSCs. This last strategy, which is based on FG enrichment with AD-MSCs, has been used and has achieved effective results in plastic surgery and for correcting breast soft tissue defects. These procedures are based on the minimal manipulation of fatty tissue via centrifugation, filtration, or enzymatic digestion using human collagenases. A recent study compared breast augmentation results obtained in patients suffering from breast hypoplasia treated with a prosthesis with those obtained in patients treated with FG enriched with AD-MSCs. The study confirmed the safety and effectiveness of both the prostheses and AD-MSC-enhanced FG in the treated case series; however, a decreased scar burden with more natural aesthetic results was observed in the group treated with FG [10].

The main limitations of FG are its resorption and its controversial relationship with breast cancer in obese patients [10]. Techniques based on FG (not-enriched FG or FG enriched with AD-MSCs) did not represent a significant risk factor for tumor recurrence, as confirmed in recent research [10]. Additionally, an innovative strategy used during conservative mastectomies and pre-pectoral breast reconstruction and based on titanium mesh that can also be used in combination with FG has been described, confirming its oncological safety [11].

The clinical improvements obtained by FG were related to the AD-MSCs contained in the stromal vascular fraction (SVF) [10,12] and consisted of a mixture of pericytes, leukocytes, and endothelial and smooth muscle cells. Additionally, FG contains several cells (AD-MSCs and adipocytes), extracellular matrices (ECMs), nerves, and vessels [12]. For the reasons mentioned above, FG may be considered as a biologically active tissue with regenerative properties when it is injected directly into skin wounds and soft tissue defects and as a scaffold when it is enriched with AD-MSCs [12]. 

FGs may act as scaffolds for AD-MSCs, representing a biological matrix (cellular and extracellular) in which these cells can be incorporated, resulting in improved healing time as well as improved scar signs and symptoms when using an autologous regenerative approach. The percentage of AD-MSCs contained in SVF varies depending on the extraction procedure (enzymatic digestion vs. mechanical manipulation based on centrifugation and filtration). Still, it is greater than the percentage observed in classic FG [10,12].

The data analyzed in this Special Issue provide indications of the positive effects of AD-MSCs in different fields, including plastic surgery.
